# Meetings of Societies

**Published:** 1930

**Authors:** 


### Meetings of Societies.

### Bristol Medico-Chirurgical Society.

The sixth meeting of the session was held at the University on Wednesday, 12th March, 1930, the President in the chair.

MR. S. V. STOCK then opened a discussion on "**The Evaluation and Treatment of the Bad Operation Risk.**"

DR. R. C. CLARKE then read a paper on "**The Atonic Child.**"

The seventh meeting of the session was held at the University on Wednesday, 9th April, 1930, the President in the chair.

MR. A. D. FRASER gave a paper on "**The Cause of Acute Rheumatic Fever and the Relationship of the Organisms to Chronic Rheumatism and Cancer.**"

The last meeting of the session was held at the University on Wednesday, 14th May, 1930, the President in the chair.

DR. J. V. BLACHFORD gave a paper on "**Epilepsy and its Relation to Mental Diseases.**"

